# The Role of Even-Skipped in *Drosophila* Larval Somatosensory Circuit Assembly

**DOI:** 10.1523/ENEURO.0403-21.2021

**Published:** 2022-02-02

**Authors:** Zarion D. Marshall, Ellie S. Heckscher

**Affiliations:** 1 Department of Molecular Genetics and Cell Biology, University of Chicago, Chicago, IL 60637; 2 Institute for Neuroscience, University of Chicago, Chicago, IL 60637

**Keywords:** embryo, larva, mechanosensation, proprioception, somatosensation

## Abstract

Proper somatosensory circuit assembly is critical for processing somatosensory stimuli and for responding accordingly. In comparison to other sensory circuits (e.g., olfactory and visual), somatosensory circuits have unique anatomy and function. However, understanding of somatosensory circuit development lags far behind that of other sensory systems. For example, there are few identified transcription factors required for integration of interneurons into functional somatosensory circuits. Here, as a model, we examine one type of somatosensory interneuron, Even-skipped (Eve) expressing laterally placed interneurons (ELs) of the *Drosophila* larval nerve cord. Eve is a highly conserved, homeodomain transcription factor known to play a role in cell fate specification and neuronal axon guidance. Because marker genes are often functionally important in the cell types they define, we deleted *eve* expression specifically from EL interneurons. On the cell biological level, using single neuron labeling, we find *eve* plays several previously undescribed roles in refinement of neuron morphogenesis. Eve suppresses aberrant neurite branching, promotes axon elongation, and regulates dorsal-ventral dendrite position. On the circuit level, using optogenetics, calcium imaging, and behavioral analysis, we find *eve* expression is required in EL interneurons for the normal encoding of somatosensory stimuli and for normal mapping of outputs to behavior. We conclude that the *eve* gene product coordinately regulates multiple aspects of EL interneuron morphogenesis and is critically required to properly integrate EL interneurons into somatosensory circuits. Our data shed light on the genetic regulation of somatosensory circuit assembly.

## Significance Statement

In general, *even-skipped* (*eve*) genes are considered neural cell fate determinants. Here, we show that *eve* gene expression is required for refinement of axon and dendrite morphogenesis and for proper functional integration of neurons into somatosensory circuits. Thus, *eve* coordinately regulates multiple terminal neuronal features of a class of Eve-expressing interneurons, raising the possibility that, in other neuronal contexts, *eve* genes regulate a similar suite of features. Our study pushes the understanding of *eve* beyond the level of neuron morphology to the levels of circuit physiology and whole animal behavior. It thereby provides an updated understanding of *eve* in development. Further, our data identify *eve* as a genetic entry point for future study of sensorimotor circuit assembly in *Drosophila*.

## Introduction

Proper assembly of somatosensory circuits is critical for perception and movement (Zeilig et al., 2012). During somatosensory circuit development, interneurons wire up in precise patterns both with sensory neurons and with other CNS neurons (Kohsaka et al., 2017; Clark et al., 2018; D’Elia and Dasen, 2018). This is a multistep process involving, first, cell fate specification; then, axon outgrowth and dendrite morphogenesis; and finally, functional integration of neuronal inputs and outputs into the circuit. However, in comparison to other sensory systems (e.g., visual and olfactory), the assembly of the somatosensory system is still poorly understood (Meng and Heckscher, 2021).

The *Drosophila* larval nerve cord is an excellent system to study somatosensory circuit development. The organization of *Drosophila* and vertebrate somatosensory circuits is similar. For example, in both, a diversity of sensory neurons project axons to distinct dorsal-ventral regions (or laminae) in the CNS (Rexed, 1952; Zlatic et al., 2009). Genetically-defined subtypes of somatosensory interneurons synapse with specific sensory neurons, and those interneurons contribute to either local reflex circuits or send information to the brain (Heckscher et al., 2015; Lai et al., 2016; Wreden et al., 2017). Therefore, principles uncovered in studies of *Drosophila* have the potential to be broadly relevant to vertebrate somatosensory circuit development.

In *Drosophila*, several aspects of somatosensory circuit development are well understood. In the peripheral nervous system (PNS), specific transcription factors that regulate sensory neuron dendrite morphogenesis have been identified (Zlatic et al., 2003; Parrish et al., 2006; Hattori et al., 2007). In the CNS, early cell fate specification and the transcriptional regulation of axon guidance have been characterized (Santiago and Bashaw, 2014; Stratmann et al., 2019). However, most studies of neurons in the *Drosophila* CNS have focused on experimentally accessible motor neurons. Motor neurons are fundamentally distinct from somatosensory interneurons. Axons of motor neurons exit the CNS, and in general, dendrites of motor neurons do not get direct synaptic input from sensory neurons (Couton et al., 2015). In *Drosophila*, there remain large gaps in our understanding of the genetic control of somatosensory interneuron morphogenesis, specifically in control of dendrite morphology and circuit integration. Furthermore, it is unclear to what extent multiple terminal features, such as axon and dendrite morphology, are coordinately controlled by single transcription factors (Kurmangaliyev et al., 2019).

In this study, as a model, we focus on *Drosophila* larval laterally placed interneurons (ELs). ELs are named for their expression of the transcription factor, Even-skipped (Eve) and their lateral cell body position. EL interneurons all process somatosensory stimuli and are necessary for normal behavior (Heckscher et al., 2015). We focus on ELs because in comparison to other *Drosophila* somatosensory interneurons, the developmental origins of ELs are known, and reagents to label ELs in embryos and larvae are available (Fujioka et al., 1999). To study the transcriptional control of EL development, we took a candidate gene approach focusing on the role of *eve*. *eve* encodes a conserved homeobox transcription factor. *eve* or its homologs are expressed in neurons in animals across phyla (Ferrier et al., 2001; Heckscher et al., 2015). We reasoned that *eve* is likely to play an important role in ELs because cell type-specific transcription factor genes often are important in the cell types they define. Further, in general, *eve* and its homologs play roles in cell fate specification and axon guidance (Doe et al., 1988; Landgraf et al., 1999; Moran-Rivard et al., 2001; Esmaeili et al., 2002; Fujioka et al., 2003; Pym et al., 2006; Zarin et al., 2014; Juárez-Morales et al., 2016). Here, we show *eve* gene expression is required for multiple aspects of terminal neuronal development and for proper functional integration of ELs into sensorimotor circuits. Specifically, *eve* regulates neurite branching, axon extension, dendrite positioning, formation of functional inputs, and mapping of functional outputs, as well as whole animal behavior. Thus, we uncover several, previously undescribed roles for *eve* in neuronal physiology, and we identify *eve* as a transcriptional regulator of *Drosophila* somatosensory circuit assembly.

## Materials and Methods

### Fly genetics

Standard methods were used to propagate fly stocks. Unless otherwise noted, larvae were raised at 25°C and fed yeast paste containing water and yeast (5:3 ratio by weight). For optogenetics experiments, yeast paste containing 100-μl all trans retinal (ATR) was used. For a list of stocks used in this study see Table 1.

**Table 1:** Methods used to label EL interneurons

		Labeling method			
Genotype		Anti-Eve	*EL-GAL4*	*UAS-FLP, actin-FRT-stop-* *FRT-GAL4;;EL-GAL4*	*11F02-GAL4*
Wild type	Neurons labeled	EL interneurons, Motor neurons	EL interneurons	EL interneurons	Late-born ELs, other neurons
	Stages	Neuron birth to larval L3	Embryos stage 14 to larval L2	Embryo stage 15 to larval L3	Embryo stage 15 to larva
EL eve mutant/ EL eve mutant	Neurons labeled	Motor neurons	EL interneurons	EL interneurons	Late-born ELs, other neurons
	Stages	Neuron birth to larval L3	Embryo stage 14 to stage 15	Embryo stage 15 to larva L3	Embryo stage 15 to larva
EL eve mutant/ *eve(5)*	Neurons labeled	EL interneurons, Motor neurons	EL interneurons	N/A	N/A
	Stages	Neuron birth to larval L3	Embryo stage 14 to larval L2	N/A	N/A

### Species sex

In these experiments, embryos and early-stage larvae were used. At these developmental stages, flies have no distinguishing sexual characteristics. Thus, all experiments were conducted in a manner that was blind to sex.

### Immunostaining

We used standard methods (Meng et al., 2019, 2020). Larval brains were pulled at ambient temperature within a 7-min window. Brains were fixed in freshly prepared 1× phosphate buffered saline containing 4% formaldehyde for 7–10 min. Many primary antibodies were obtained from Developmental Studies Hybridoma Bank, created by the NICHD of the NIH and maintained at The University of Iowa, Department of Biology. See Table 1 for primary antibodies. Secondary antibodies were from Jackson ImmunoResearch and were used according to manufacturer’s instructions. Images were acquired on a Zeiss 800 confocal microscope with 40× objective. Embryos were staged for imaging based on standard morphologic criteria.

### Four ways to label ELs interneurons

In this study, we use four ways to label ELs (Table 2).

**Table 2:** Antibodies, fly lines

Antibodies (concentration) source (catalog number)	
Rabbit-Eve (1:500)	Ellie Heckscher, University of Chicago (1432p)
Mouse-Eve (preabsorbed 4 μg/ml)	Developmental Studies Hybridoma Bank (DSHB; 2B8)
Mouse-FasII (1:100)	DSHB (1D4)
Rat-Flag (1:300)	Novus (catalog #NBP1-06712; RRID: AB_1625981)
Mouse-HA (1:300)	BioLegend (catalog #901501; RRID: AB_2565006)
Chicken-V5 (1:300)	Bethyl (catalog #A190-118A; RRID: AB_66741)
Chicken-GFP (1:500)	Aves & Abcam (AB_2307313 ab13970)
Guinea Pig-Hb9 (1:1000)	Heather Broihier, Case Western
Mouse-Eagle (1:50)	Abcam (ab 2013237)
Mouse-AbdA/Ubx (1:400)	DSHB (FP6.87)
Mouse-AbdB (1:400)	DSHB (1A2E9)
Mouse-En (1:5)	DSHB (4D9)
Rabbit-Cas (1:500)	Chris Doe, University of Oregon
Mouse-Cut (1:50)	DSHB (2B10)
Rat-Dpn (1:50)	Chris Doe, University of Oregon
Mouse-Islet (1:10)	DSHB (40.3A4)
Guinea Pig-Kruppel (1:1000)	John Reintz, University of Chicago
Rabbit-Nab (1:1000)	Chris Doe, University of Oregon
Guinea Pig-Knot (1:1000)	Adrian Moore, RIKEN
Rat-Pdm2 (1:100)	Chris Doe, University of Oregon
Mouse-Svp (1:500)	DSHB (6F7)
Rat-Zfh2 (1:200)	Chris Doe, University of Oregon
Mouse-Antp (1:400)	DSHB (8C11)
Mouse-Repo (1:10)	DSHB (8D12)
Fly lines	
*11F02-gal4*	Bloomington Drosophila Stock left (BDSC): 9828
*actin-FRT.stop-Gal4*	BDSC: 4779
*UAS FLP, BRP-frt-stop-frt-V5-2A-LexA*	BDSC: 55749
*Df(2R) eve*	BDSC: 1545
*eve(3)*	BDSC: 299
*eve(5)*	BDSC: 4084
*UAS-Chrimson.mVenus*	BDSC: 5535
*UAS-FLP*	BDSC: 8208
*UAS(FRT.stop)myr::smGdP-HA,UAS(FRT.stop)myr::smGdP-V5,* *UAS(FRT.stop)myr::smGdP-FLAG; FLPG5.PEST*	BDSC: 64085
*UAS-GCaMP6m*	BDSC: 42786
*UAS-myr-GFP*	BDSC: 32197
*EGN92, ΔEL_B*	Fujioka et al. (2003)
*EL-GAL4*	Fujioka et al. (1999)
*UAS-RPR, UAS-HID*	Heckscher et al. (2015)

#### Eve antibody staining

In wild-type, anti-Eve labels motor neurons and EL interneurons. In EL eve mutants, anti-Eve staining is lost from the ELs. Notably, in EL eve mutants in trans to a hypomorphic *eve* allele, *eve(5)*, which generates a truncated Eve protein, anti-Eve can be used to track ELs.

#### EL-GAL4

In wild-type, *EL-GAL4* expresses in ELs from the middle of embryogenesis until the middle of larval development. However, in EL eve mutants, *EL-GAL4* drives only a pulse of gene expression. Thus, in ELs, Eve is dispensable for initiation of *EL-GAL4* expression, but is required to maintain *EL-GAL4* expression.

#### *EL-GAL4* with a permanent labeling cassette

To positively mark ELs in an EL eve mutant background using *EL-GAL4*, we added a FLP-based permanent labeling cassette (*UAS-FLP, actin-FRT-Stop-FRT-GAL4*).

#### 11F02-GAL4

In wild-type, *11F02-GAL4* drives expression in the late-born subset of ELs, as well as a few other uncharacterized neurons (Heckscher et al., 2014). *11F02-GAL4* expression is unchanged in EL eve mutants.

### Larval behavior

L1 larvae were collected from 0 to 6 h after hatching. All larvae were rinsed in mesh chambers under dH20 until they were freed from food debris. Collected larvae were placed on 5-mm-thick 2% agarose gels set in 5.5-cm-wide Petri dishes at least 15 min before recording. All recordings were done at 22–25°C. Five to 30 larvae were allowed to freely crawl on a gel per recording. For pinching assay, pinches were delivered to the side of the larval body wall with a pair of forceps. A larva was considered to be rolling if the trachea disappeared under one side of the larva body and reappeared on the other side. Note that this criterion for a roll is not based on larval speed. For hunching assay, a vibration was delivered to the larvae using the speaker and sound described below. Hunches were considered to be a shortening of the distance between the head and body center associated with a pause in crawling, but not a head turn. For left-right asymmetry and crawling speed assays, larvae were recorded in our custom-built behavior rig (Wreden et al., 2017). Images were acquired at 10 frames per second. For optogenetic experiments, all larvae were recorded 48–54 h after hatching. A total of 10–100 larvae were placed on a rectangular 5-mm-thick agarose gel at least 15 min before recording. Recordings began with a 30-s period of no stimulus followed by a 30-s period of stimulating light and ending with a final 30-s period of no stimulus. For further setup details, please refer to Wreden et al. (2017).

Larvae were tracked using FIMTracker (Risse et al., 2017) software using default parameters including five spline points, this includes centroid, head, and tail points, plus two other points, one halfway between head and centroid and one halfway between the tail and centroid. Larvae were discarded from analysis if (1) the larva’s track was <300 frames; (2) the larva was improperly masked; or (3) if occasionally the larva collided with another larva and it was not rectified by thresholding. Tracks were then analyzed using custom scripts (Heckscher et al., 2015) on MATLAB (MathWorks). Speeds were calculated using the center spline point over a 10 frame (1-s) window. Statistical tests were performed using Prism 9 software (GraphPad).

### Calcium imaging

For calcium imaging experiments, all larvae were within 6 h of age on the day of recording and collected 48–54 h after hatching. Larvae expressing GCaMP6m (*UAS-FLP, act5C-FRT.stop-GAL4; ΔEL, Df(2R)eve/+; EL-GAL4/UASGCaMP6m* or *UAS-FLP, act5C-FRT.stop-GAL4; ΔEL, Df(2R)eve/ΔEL, Df(2R)eve; EL-GAL4/UAS-GCaMP6m*) were rinsed with water and placed ventral side up on agarose pads with a 22 × 22 mm coverslip placed on top. Pads were made by pouring 3% agarose into a well. Recordings began with a 30-s period of no stimulus followed by a 30-s period of sound stimulus and ending with a final 30-s period of no stimulus. A Visaton FR12, 4 Ohm speaker (5 inches in diameter) and a PYLE PCA2 stereo power amplifier was used to project sound. For further details, refer to Wreden et al., 2017. Images were acquired on a Zeiss LSM 800 confocal microscope using 0.1–0.2% 488-nm laser power with the pinhole entirely open. Images were acquired at three frames per second using a 10× (0.3 NA) or 20× (0.8 NA) objective. The calcium signal was continuously collected before, during, and after the stimulus. Extracting changes in GCaMP6m fluorescence amplitude was done using Fiji as in Wreden et al. (2017). A region of interest (ROI) that included the larval nerve cord was manually drawn, and the mean fluorescence within the ROI was calculated for each time point.

### Single neuron labeling

We labeled single neurons using MultiColor FLP Out (MCFO; Nern et al., 2015). MCFO stochastically labels the membranes with epitopes in cells within a GAL4 pattern. MCFO uses FLP recombinase, which we also use to label larval EL neurons in EL eve mutants (Table 2). So, we cannot use these reagents together. Here, for MCFO, we used *11F02-GAL4,* which is expressed in late-born ELs and three poorly characterized non-EL interneurons directly adjacent to ELs (Heckscher et al., 2014; Wreden et al., 2017). For *11F02-GAL4* to be useful, we needed to distinguish between ELs and non-ELs in wild-type and EL eve mutant backgrounds. In both backgrounds, we performed single neuron MCFO labeling. In both backgrounds, we can identify three non-EL neurons based on morphology, which we call: 11F02d, 11F02m, and 11F02z. We conclude that loss of Eve from ELs does not have gross nonautonomous effects on non-EL neurons. We also conclude that we can distinguish between EL and non-EL neurons in EL eve mutants. To obtain single cell clones, adult flies were allowed to lay eggs for 24 h on apple juice caps. Caps were heat shocked in a water bath at 37–39°C for 15–30 min and incubated at 25°C for 4–5 h. First instar larvae were dissected. Their brains were stained for HA, Flag, and V5 epitopes to visualize single cell clones. Larvae were also stained for Eve protein to confirm the identity of each single cell clone as an EL, and to assign segmental identity to each clone. Clones were imaged on a Zeiss 880 or 800 confocal microscope. We generated >115 single-cell clones. Among these clones, we saw each neuronal morphology in a minimum of two independently derived clones (i.e., larvae). Each clone was analyzed in dorsal and posterior views.

## Results

### In EL eve mutants, EL interneurons lack *eve* expression

**Figure 1.**
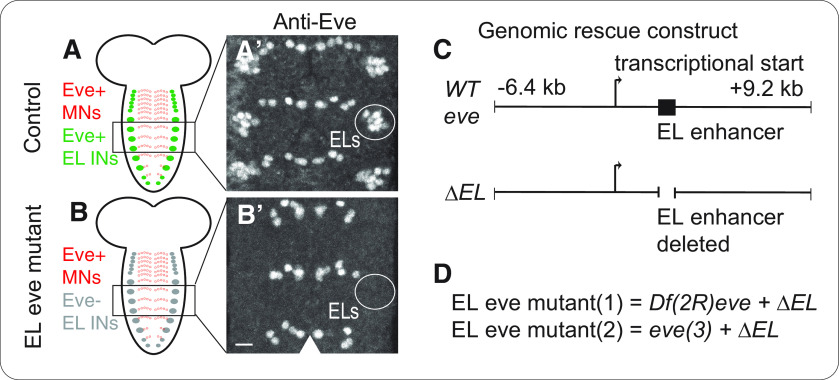
In EL eve mutants, EL interneurons lack Eve expression. ***A***, ***B***, Images of Eve expression in the nerve cord of *Drosophila* embryos. ***A***, In control, each segment has Eve(+) motor neurons (MNs; red) and Eve(+) EL interneurons (INs; green). ***B***, In EL eve mutants, Eve is selectively lost from EL, but neurons themselves remain (Fujioka et al., 2003). Images show Eve expression in three segments of the *Drosophila* nerve cord of stage 16 embryos. Anterior is up with scale bar of 15 μm. Position of EL interneurons in one hemisegment is circled. Midline is marked by an arrowhead. ***C***, ***D***, Schematics of genomic constructs that rescue *eve* expression. ***C***, The top line shows a wild-type “WT eve” genomic DNA fragment (EGN92; Fujioka et al., 2003), which contains all known *eve* coding and regulatory sequences. The bottom line represents the “ΔEL” rescue construct, which is identical to WT *eve* construct except it lacks the EL enhancer (EGN92 ΔEL; Fujioka et al., 2003). ***D***, Two different *eve* null alleles, *Df(2R)eve* and *eve(3)*, are rescued with the ΔEL construct, referred to EL eve mutant (1) and EL eve mutant (2), respectively. Genotypes: control is Δ*EL, Df(2R)eve/+* and EL eve mutant is Δ*EL, eve(3)/*Δ*EL, Df(2R)ev*e.

The objective of this study was to determine the role of the transcription factor Eve in EL somatosensory interneurons. *eve* is an essential gene in *Drosophila*. It is expressed in stripes in early embryos, in other developing tissues, as well as in a subset of motor neurons and EL interneurons (Frasch et al., 1987; Heckscher et al., 2014; Fig. 1*A*). In this study, we removed *eve* gene expression specifically from ELs using “EL eve mutants,” which were previously generated by Fujioka and colleagues (Fujioka et al., 2003). Briefly, a genomic construct containing all *eve* regulatory elements was generated (Fig. 1*C*). This construct rescues *eve* null mutants to viability, and *eve* is expressed at normal levels and in normal locations (Fujioka et al., 2003). Fujioka and colleagues deleted from the construct a regulatory region sufficient to drive *eve* expression in ELs, thereby making a “ΔEL” construct (Fig. 1*C*; Fujioka et al., 1999, 2003). When *eve* null alleles are rescued with ΔEL, *eve* is expressed at normal levels and locations everywhere except ELs interneurons, which completely lack Eve protein (Fig. 1*B*; Fujioka et al., 2003).

### *eve* regulates multiple aspects of interneuron morphogenesis

*eve* or its homologs (e.g., *evx* genes) often regulate neuronal axonal pathfinding. For example, in mouse spinal cord, *evx1* is required for V0v interneurons to send axons across the midline (Moran-Rivard et al., 2001). In *Drosophila* or *Caenorhabditis elegans* motor neurons, loss of *eve* genes results in dramatic changes in axon trajectories (Esmaeili et al., 2002; Fujioka et al., 2003). In contrast, in Zebrafish, *evx1/2*(–) V0v interneurons have normal axonal morphology (Juárez-Morales et al., 2016). These data demonstrate that *eve* plays a context dependent role in axons and raise the question to what extent *eve* regulates EL interneuron axon morphogenesis in *Drosophila* larvae.

**Figure 2.**
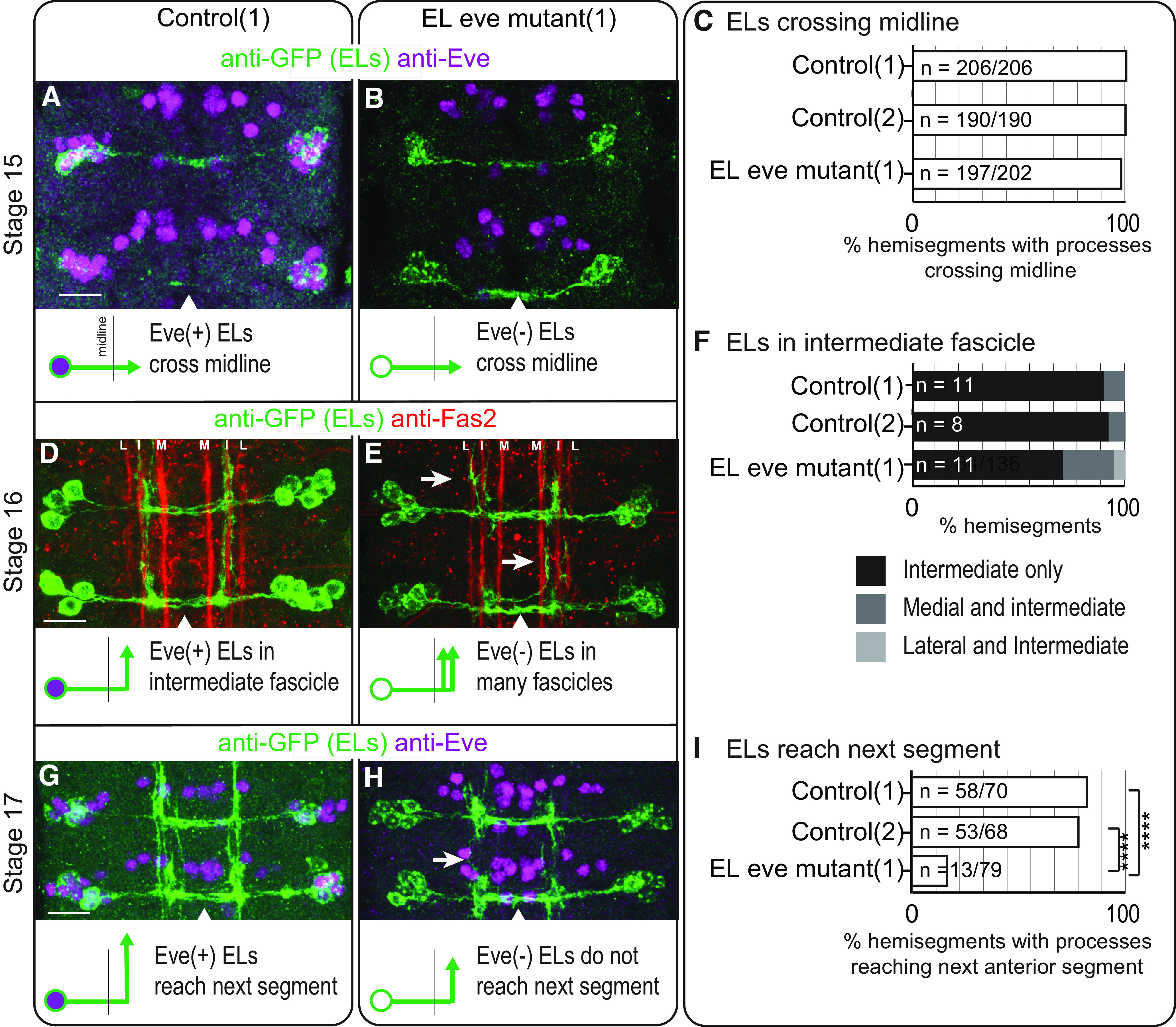
In embryos, *eve* expression is required for proper medial-lateral and anterior-posterior neurite positioning of EL interneurons. ***A–C***, Images and quantification of stage 15 embryos with EL interneurons extending axons across the midline. ***A***, ***B***, In control and EL eve mutants, ELs extend across the midline. Two abdominal segments are shown with midline noted as an arrowhead. Anterior is up and scale bars are 10 μm. Below each image is an illustration of the phenotype. ***C***, For this quantification, *n* = number of hemisegments with EL processes crossing the midline over the total number of hemisegments scored. ***D–F***, Images and quantification of stage 16 embryos with defects in medial-lateral positioning of EL neurites in EL eve mutants. ***D***, In control, ELs project toward the anterior mainly along the intermediate fascicle. ***E***, In EL eve mutants, ELs project in additional fascicles (arrows; L = lateral fascicle, I = intermediate, M = medial). ***F***, For this quantification, *n* = number of hemisegments scored. ***G–I***, Images and quantification of stage 17 embryos, with defects in anterior-posterior positioning of EL neurites in EL eve mutants. ***G***, In control, ELs extend neurites to the next anterior segment. ***H***, In EL eve mutants, neurites do not extend to the next segment (arrow). ***I***, For this quantification, *n* = number of hemisegments with processes reaching the next segment over the total number of hemisegments scored; chi-squared test, ****p < 0.0001. Genotypes: control 1 is *EL-GAL4/UAS-myr-GFP*; control 2 is *UAS-FLP, act5C-FRT.stop-GAL4;;EL-GAL4/UAS-myr-GFP*. EL eve mutant (1) is *UAS-FLP, act5C-FRT.stop-GAL4;* Δ*EL, Df(2R)eve/*Δ*EL, Df(2R)eve; EL-GAL4/UAS-myr-GFP*.

In embryos, we characterized EL morphology by expressing a membrane localized GFP. In stage 15 embryos, in both control and EL eve mutants, EL axons cross the midline (Fig. 2*A–C*). In stage 16 embryos, we additionally stained with anti-Fas2 to visualize three fascicles in the neuropile—lateral, intermediate, and medial (Landgraf et al., 2003). In control, after midline crossing, most ELs grow laterally until reaching the intermediate fascicle and extend toward the anterior. In a subset of segments, ELs extend to the anterior along both intermediate and medial fascicles (Fig. 2*D*,*F*). In EL eve mutants, however, a larger proportion of ELs extend along both the intermediate and medial fascicles. Further, in some segments, ELs project along the intermediate and lateral fascicles, a phenotype never observed in controls (Fig. 2*E*,*F*). In stage 17 embryos (the final stage of embryogenesis), in control, ELs project so far to the anterior that they reach the next segment, making a ladder like pattern (Fig. 2*G*). In contrast, in EL eve mutants, only a small proportion of Eve(–) ELs reach the next segment (Fig. 2*H*,*I*).

**Figure 3.**
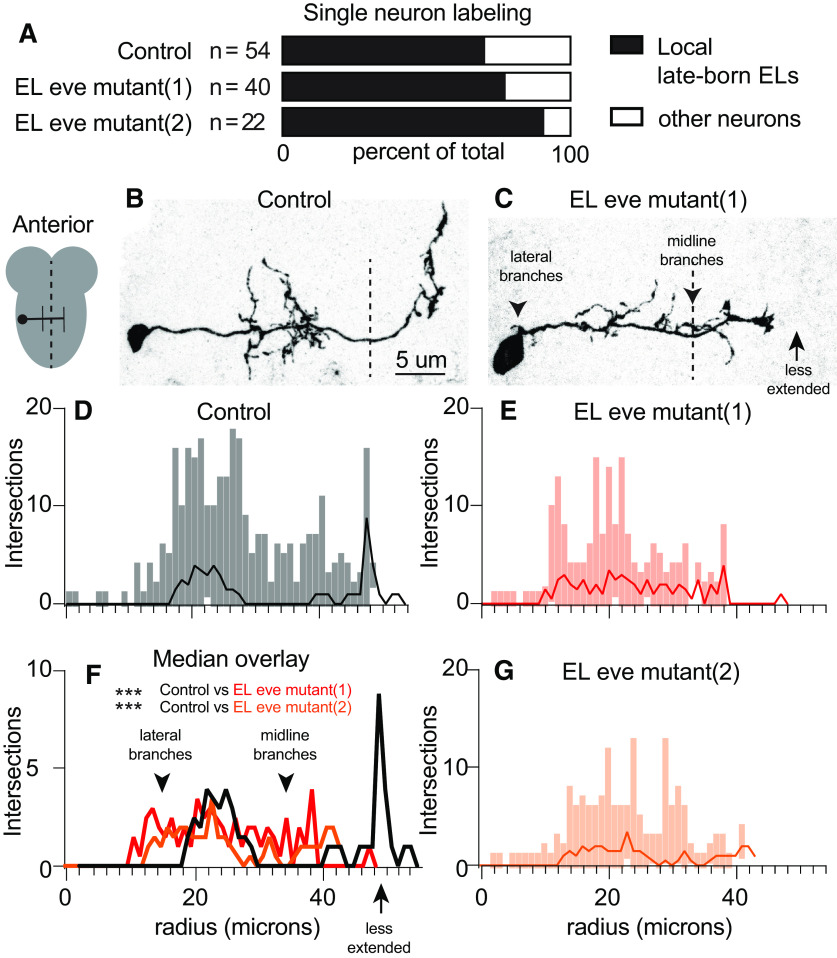
In larvae, Eve(–) ELs have excessive branching off the main neurite and diminished axon extension. ***A***, Quantification of labeled neurons. In all genotypes, the most numerous type of singly-labeled neurons are local, late-born ELs. *n* = total number of singly-labeled neurons for each genotype. ***B***, ***C***, Images of singly-labeled ELs. *Drosophila* neurons are pseudo-unipolar. ***B***, In control, dendrites are located ipsilateral to the soma. On the contralateral side, axons turn to the anterior and form branches where output synapses are found (Heckscher et al., 2015). ***C***, In EL eve mutants, there is excessive branching off the main neurite (arrowheads), and the axon is less extended (arrow). Anterior is up with a scale bar of 5 μm. Dashed line indicates midline. ***D–G***, Quantifications of neuron morphology. For each plot, the *x*-axis is the radius of a concentric circle centered on the EL soma. The *y*-axis is number of times a singly-labeled EL intersects a circle. Median (dark line) and range (lighter bars) are shown. ***F***, Control is black; red and orange lines are EL eve mutants (1) and (2), respectively. Wilcoxon test, ***p < 0.0001. Genotypes: control is *11F02-GAL4/UAS-MCFO*. EL eve mutant (1) is Δ*EL, Df(2R)eve/*Δ*EL, Df(2R)eve; 11F02-GAL4/UAS-MCFO.* EL eve mutant (2) is Δ*EL, Df(2R)eve/*Δ*EL, eve(3); 11F02-GAL4/UAS-MCFO*.

At larval stages, ELs have mature morphologies and are incorporated into functioning somatosensory circuits. We labeled individual ELs, using MCFO, which allows us to determine which part of the EL, axon, dendrite, or both, is impacted by loss of *eve* expression (Nern et al., 2015). We restricted our analysis to local, late-born ELs, because they were most abundantly labeled in our dataset (Fig. 3*A*). First, we focused our analysis along medial-lateral and anterior-posterior axes because in Figure 2 we detected defects along these axes. We used Sholl analysis to count the number of intersections between an EL neurite and concentric circles with increasing radii (1-μm intervals) centered on the soma (Sholl, 1953). We plotted the median, minimum, and maximum number of intersections versus circle radius to generate a description of arborization. In control, local, late-born ELs have two sets of branching neurites off the main neurite. Ipsilaterally (same side) dendritic neurites are found ∼20 μm from the soma. Contralaterally (opposite side) axonal neurites branch ∼50 μm from the soma (Fig. 3*B*,*D*). In EL eve mutants, neurites of Eve(–) ELs branched excessively off the main neurite near the soma and in the midline, which is never seen in wild type (Fig. 3*C*, arrowheads). Furthermore, Eve(–) EL axons are less extended, branching at ∼40 μm from the soma (Fig. 3*C*, arrow). These changes are reflected in a statistically significant difference in the distribution of intersections (Fig. 3*F*).

**Figure 4.**
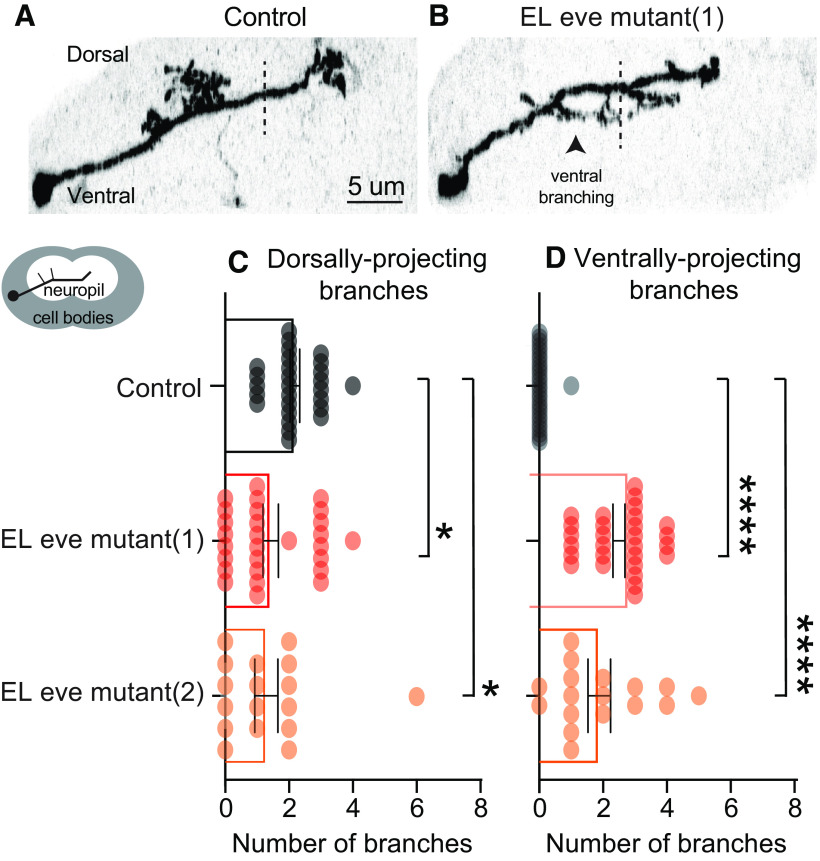
Eve(–) ELs have mispositioned dorsal-ventral dendrites. ***A***, ***B***, Images of singly-labeled ELs in side view. ***A***, In control, ELs have ipsilateral dendrites that project dorsally. ***B***, In EL eve mutants, many branches project ventrally. These are the same neurons as in Figure 3*B*,*C*, except here dorsal is up. ***C***, ***D***, Quantification of dendrite orientation. The number of branches pointing dorsally or ventrally is plotted with each dot representing a single neuron. Bars show average, and whiskers show standard deviation. ANOVA with Dunnett’s multiple comparison; *p < 0.05 and ****p < 0.0001.

Next, in the same set of larval clones as shown in Figure 3, we characterized ELs along the dorsal-ventral axis. For somatosensory interneuron dendrites, positioning along the dorsal-ventral axis is particularly important because different sensory neurons axons project to different dorsal-ventral domains within the CNS (Landgraf et al., 2003). Therefore, dorsal-ventral positioning of somatosensory interneuron dendrites is expected to dictate the types of sensory input received by a given interneuron. Currently, the molecular control of dorsal-ventral dendrite positioning of somatosensory interneurons is extremely poorly understood. Here, we counted the number of dorsally and ventrally projecting ipsilateral branches (i.e., dendrite). In control, nearly all Eve(+) ELs have dorsally, but not ventrally, projecting dendrites (Fig. 4*A*,*C*,*D*). In EL eve mutants, there is a significant reduction in dorsally projecting dendrites and a significant increase in ventrally projecting dendrites for Eve(–) ELs (Fig. 4*B*–*D*).

**Figure 5.**
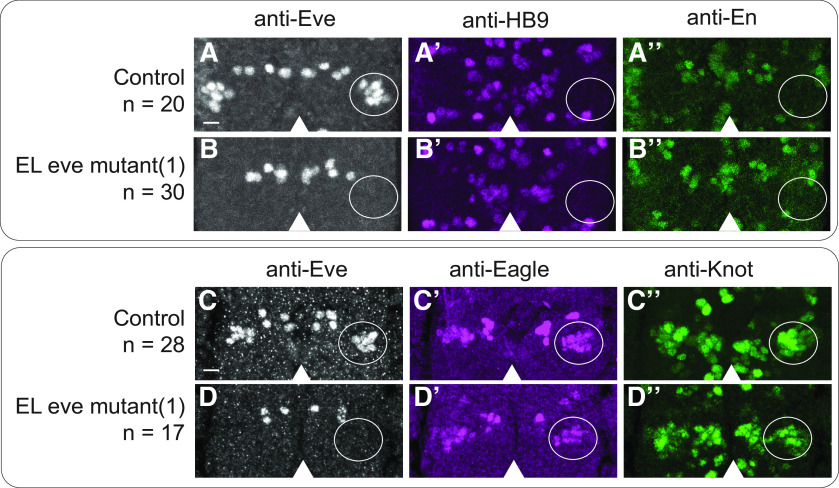
Eve(–) ELs do not derepress molecular markers. ***A–D***, Images of marker gene expression in ELs. ***A***, ***C***, In control, ELs lack expression of ventral motor neuron maker, HB9 (***A’***) and an interneuron marker En (***A’’***). ELs express both Eagle (***C’***) and Collier (***C’’***). ***B***, ***D***, In EL eve mutants, there is no change in marker gene expression. Representative segments of stage 16 embryos. Anterior is up with a scale bar of 5 μm. Arrowhead shows midline. Area containing EL neurons is circled. *n* = number of hemisegments scored. Each row shows separate image channels of the same co-stained stained embryo. Genotypes: control is *wild-type* and EL eve mutant(1) is *ΔEL, Df(2R)eve/ΔEL,* Df(2R)eve.

Finally, we asked whether loss of *eve* expression transforms the ELs into another interneuron type. To do so, we took both anatomic and genetic approaches. Anatomically, we mined the *Drosophila* larval connectome (Ohyama et al., 2015). We looked at each local neuron in the first abdominal segment, but found no neurons with morphology matching that of Eve(–) EL interneurons. Genetically, we looked for large-scale changes in gene expression, which often accompany cell fate changes. We surveyed 19 genes that were candidates to be regulated by *eve* in ELs. These candidates included genes regulated by *eve* in non-EL cell types (En, FasII, HB9, Islet; see Fig. 5*A*,*B*), genes expressed in ELs (Castor, Eagle, Zfh2, Knot, Kruppel, Nab, Pdm2, Seven-up; see Fig. 5*C*,*D*), and genes with putative Eve binding sites (Antp, AbdA/Ubx, AbdB, Cut, Dpn, Repo; Broihier and Skeath, 2002; Pym et al., 2006). There are no obvious changes in expression for any of these genes in Eve(–) ELs. Taken together, these data support the conclusion that, in ELs, Eve is not repressing alternative interneuron fate.

In summary, we find that, in ELs*, eve* expression is not required for the initial step of EL morphogenesis—axon midline crossing. However, *eve* is required for later stages of EL morphogenesis, refining morphology in all three axes, medial-lateral, anterior-posterior, and dorsal-ventral. Further, *eve* is required in both axons and dendrites. Thus, in EL interneurons, *eve* expression coordinately regulates multiple aspects of morphogenesis. Finally, we find no evidence to support the idea that, in ELs, Eve represses alternative neuronal fates because Eve(–) EL interneurons do not resemble any other wild-type interneuron.

### Loss of *eve* expression disrupts EL somatosensory stimulus encoding

**Figure 6.**
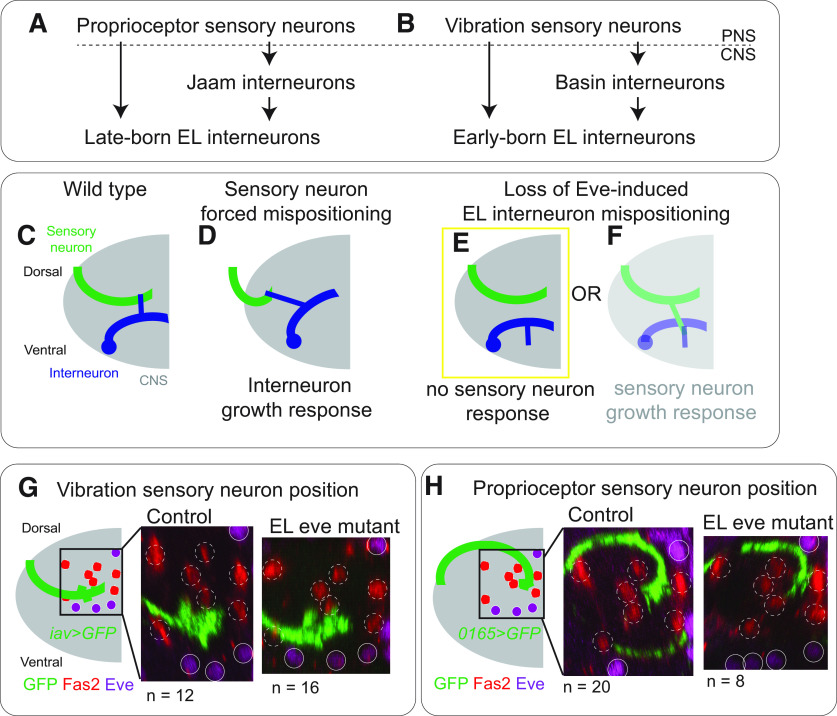
In EL eve mutants, there are no changes in sensory neurons axonal trajectories. ***A***, ***B***, Illustration of sensory inputs onto ELs. ***A***, Late-born ELs get direct input from proprioceptors and indirect input via the Jaam CNS interneurons. ***B***, Early-born ELs get direct input from mechanoreceptors (chordotonals) and indirect input via the Basin CNS interneurons. ***C–F***, Illustrations of sensory neuron-to-interneuron wiring in different genetic conditions. ***C***, In wild-type, sensory neuron axons (green) and interneuron dendrites (blue) are in close enough proximity they can form synaptic contacts. ***D***, When sensory neuron axons are genetically mispositioned, interneurons grow in response, and the two cell types continue to form synaptic contacts. ***E***, ***F***, When EL interneuron dendrites are mispositioned because of lack of Eve, sensory neurons might or might not change position in response. ***G***, ***H***, Images of vibration and proprioceptive sensory neurons arbors. ***G***, ***H***, Axonal positions are similar in control and EL eve mutants for both vibration sensitive and proprioceptive sensory neurons. Single hemisegments of an L1 larval CNS are shown with dorsal up. Eve-expressing motor neurons are shown as solid circles with a diameter of five microns. Positions of Fas2(+) fascicles are shown as dashed circles. To the left of each image is a schematic of the axon position relative to landmarks. *n* = the number of hemisegments scored. Genotypes: control in ***G*** is *iav-GAL4/UAS-myr-GFP.* EL eve mutant in ***G*** is Δ*EL, Df(2R)eve/*Δ*EL, Df(2R)eve; iav-GAL4/UAS-myr-GFP.* Control in ***H*** is *0165-GAL4/UAS-myr-GFP.* EL eve mutant in ***H*** is Δ*EL, Df(2R)eve/*Δ*EL, Df(2R)eve; 0165-GAL4/UAS-myr-GFP*.

In wild-type animals, EL interneurons encode somatosensory stimuli. Late-born ELs get direct synaptic input from proprioceptive sensory neurons and indirect proprioceptive input via Jaam interneurons (Fig. 6*A*; Heckscher et al., 2015). Early-born ELs get direct synaptic input from vibration sensitive (chordotonal) sensory neurons and indirect chordotonal input via Basin interneurons (Fig. 6*B*; Wreden et al., 2017). The observation that Eve(–) EL dendrites are mispositioned raised the possibility that *eve* is required for ELs to properly encode somatosensory stimuli (Fig. 4). We test this idea here.

Mispositioned dendrites in Eve(–) EL interneurons likely disrupt the ability of ELs to form connections with their normal sensory neuron input partners (Fig. 5*C*,*E*). Alternatively, ELs could still form synapses with input partners via a compensatory mechanism (Fig. 6*F*). It is essential to consider this alternative because a recent study revealed the existence of compensatory mechanisms in *Drosophila* sensory neuron-to-somatosensory interneuron wiring (Valdes-Aleman et al., 2021). Specifically, genetically mispositioned sensory neurons form synapses with many of their normal interneuron partners, which grow abnormally to reach the mispositioned sensory neurons (Fig. 6*D*). In control and EL eve mutants, we characterized the position of sensory neurons by labeling vibration and proprioceptive sensory neurons with *iav-GAL4* and *0165-GAL4* driving membrane GFP but find no differences in sensory neuron axon position (Fig. 6*G*,*H*).

**Figure 7.**
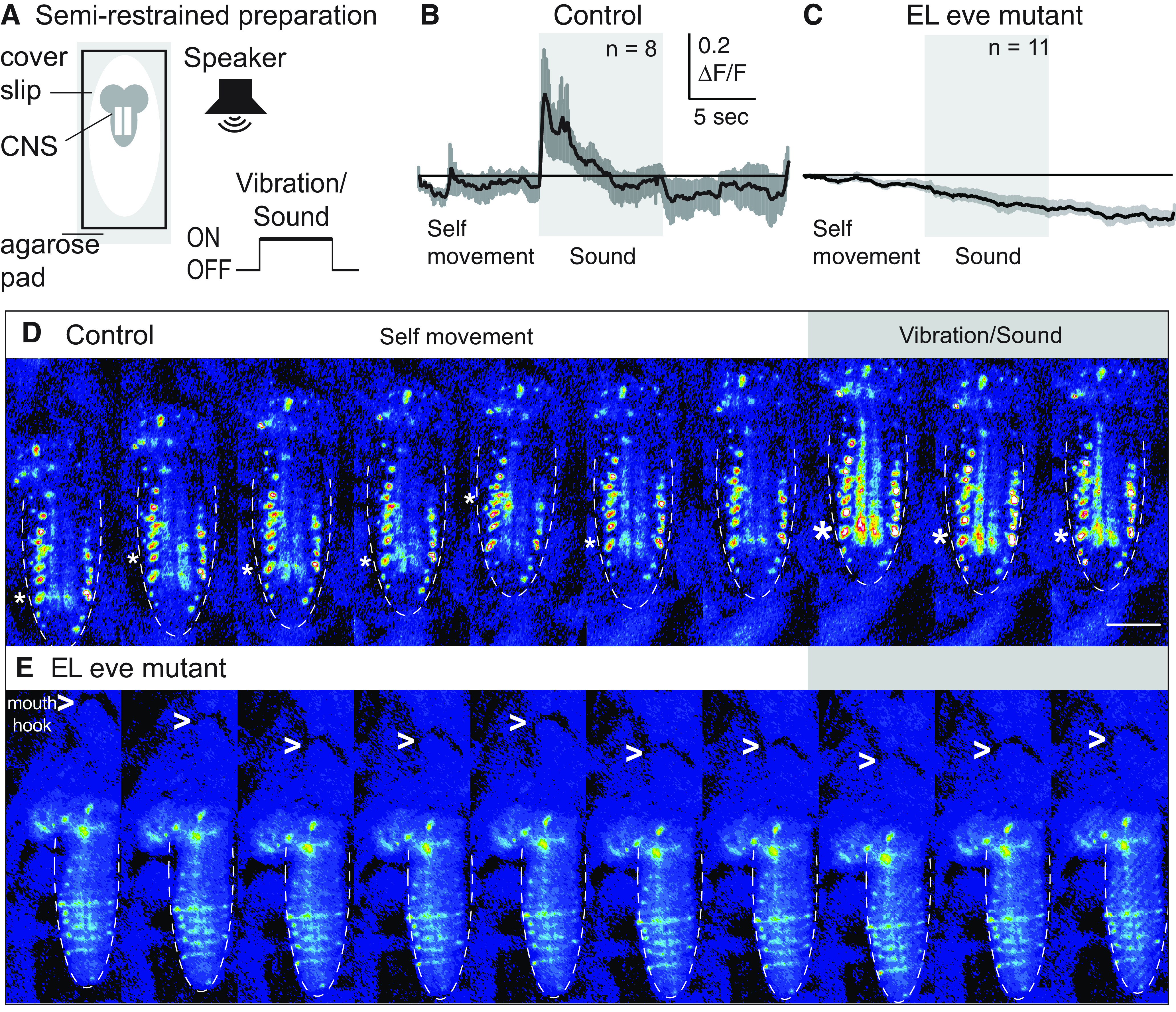
EL interneurons require *eve* expression to encode somatosensory stimuli. ***A***, Illustration of the semi-restrained preparation and stimulus protocol. Fluorescence in the CNS (gray lobed structure with two white lines representing neuropil) is recorded before, during, and after a sound is played from a speaker. ***B***, ***C***, Quantifications of EL calcium signals. ***B***, In control, EL have small amplitude, dynamic calcium signals before sound onset, which corresponds to periods of self-movement. There are large amplitude changes in EL calcium signal on sound/vibration stimuli. ΔF/F is the change in fluorescence over baseline. ***C***, In EL eve mutants, ELs do not respond to stimuli. Averages (dark line) and SEM (light line) are shown. Scale for both is shown as an inset. *n* = number of larvae recorded. ***D***, ***E***, Images from representative recordings of calcium signals in ELs. Fluorescence images are shown in pseudo-color with white/red as high fluorescence intensity and blue as low. Anterior is up with a scale bar of 100 μm. Dashed lines show the outline of the nerve cord. In ***D***, asterisk denotes region of nerve cord neuropile (central region) with increased fluorescence. In ***E***, > points to mouth hooks. Genotypes: control is *UAS-FLP, act5C-FRT.stop-GAL4;* Δ*EL, Df(2R)eve/+; EL-GAL4/UAS-GCaMP6m.* EL eve mutant is *UAS-FLP, act5C-FRT.stop-GAL4;* Δ*EL, Df(2R)eve/*Δ*EL, Df(2R)eve; EL-GAL4/UAS-GCaMP6m*.

Next, we tested the idea that *eve* expression is required for normal EL somatosensory stimulus encoding. *Drosophila* larvae are optically clear so we used calcium signals in intact larvae to monitor EL activity. Specifically, a larva expressing GCaMP6m in ELs was placed on a bed of agarose with a coverslip on top (Fig. 7*A*). In this preparation, larvae do not crawl, but they move, which stimulates proprioceptors, and sound can be used to stimulate vibration-sensitive chordotonal sensory neurons (Wreden et al., 2017). In a single recording, we imaged baseline EL fluorescence, EL activity while the sound is played, and return to baseline. In control, there are small amplitude changes in fluorescence intensity in ELs during periods of self-movement and large amplitude changes in response to vibration (Fig. 6*B*,*D*). In EL eve mutants, there is only bleaching of the calcium signal (Fig. 7*C*,*E*).

We conclude that in the absence of *eve* expression, EL interneurons no longer normally encode somatosensory stimuli.

### Upon loss of *eve* expression, EL outputs are anatomically and functionally reconfigured

EL interneurons are necessary for normal *Drosophila* larval behavior (Heckscher et al., 2015). Late-born ELs contribute to a circuit that regulates left-right symmetrical crawling, and early-born ELs contribute to a circuit that triggers fast escape rolling (Wreden et al., 2017). The observation that Eve(–) EL axons are less extended raised the possibility that EL output circuits could be reconfigured (Fig. 3).

**Figure 8.**
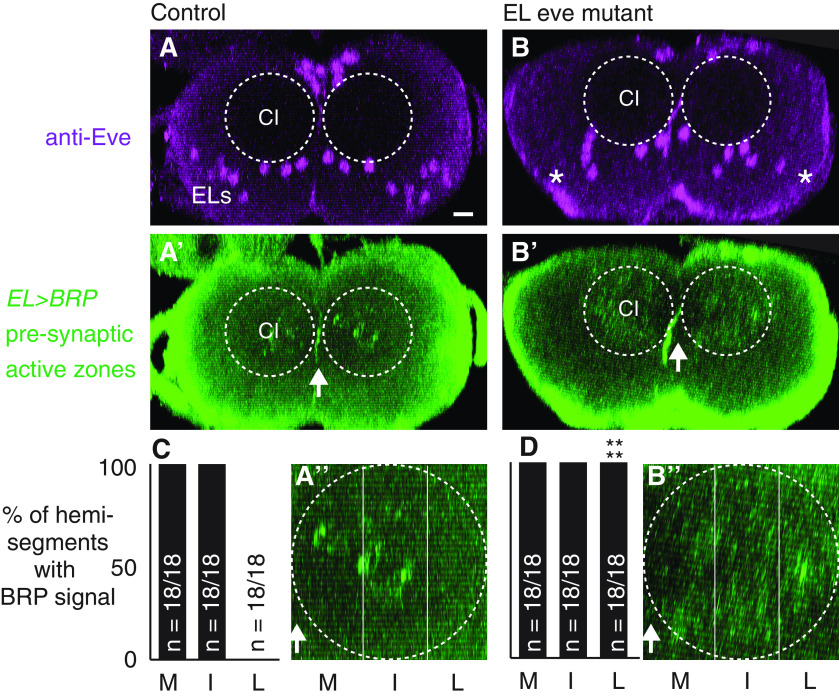
In Eve(–) ELs, output synapses are anatomically repositioned. ***A***, ***B***, Images of tagged presynaptic active zones. Eve labels ELs (“ELs” in ***A***) but not in EL eve mutants (* in ***B***). ***A’***, In control, labeled active zones (BRP) are clustered around the central intermediate Fas2(+) fascicles (CI). ***B’***, In EL eve mutants, BRP signal is diffuse throughout the entire neuropile. ***A–A’’*** are the same CNS and ***B–B’’*** are the same CNS. ***A’’***, ***B’’*** are a magnifications of the neuropile from one hemisegment in ***A’*** or ***B’***, respectively. Images are overlaid with lines showing medial (M), intermediate (I), and lateral (L) zones. Images show the CNS in cross section with dorsal up. Arrow denotes midline. Neuropile is outlined by a dashed circle. Scale bar: 10 μm. ***C***, ***D***, Quantifications of BRP signal distribution. *n* = number of hemisegments with BRP signal above background within a given region/total number of hemisegments scored. Zones scored were medial (M), intermediate (I), and lateral (L) as shown in ***A’’***, ***B’’***. Genotype: control is *UAS-FLP, act5C-FRT.stop-GAL4;* Δ*EL, Df(2R)eve/+; EL-GAL4/UAS-FLP, BRP-frt-stop-frt-V5-2A-LexA.* EL eve mutant is *UAS-FLP, act5C-FRT.stop-GAL4;* Δ*EL, Df(2R)eve/*Δ*EL, Df(2R)eve; EL-GAL4/UAS-FLP, BRP-frt-stop-frt-V5-2A-LexA.*

The specific output partners of EL interneurons are not well characterized, and so we determined the location of Eve(–) EL output synapses. In control and EL eve mutants, we visualized presynaptic nerve termini, using expression of a V5 epitope tagged Brunchpilot (BRP-V5) protein (Wagh et al., 2006; Chen et al., 2014). In control, BRP signal is concentrated in large puncta in the central intermediate and medial region of the neuropile (Fig. 8*A’*). However, in EL eve mutants, BRP signal is found diffusely throughout the neuropile (Fig. 8*B’*). To quantify this, we used Fas2 expression to subdivide the neuropile into medial (M), intermediate (I), and lateral (L) zones, and scored for BRP signal in each zone (Fig. 8*C*,*D*). We find a significant increase in lateral signal in EL eve mutants compared with control. These data suggest that Eve(–) EL interneurons output synapses are relocated.

**Figure 9.**
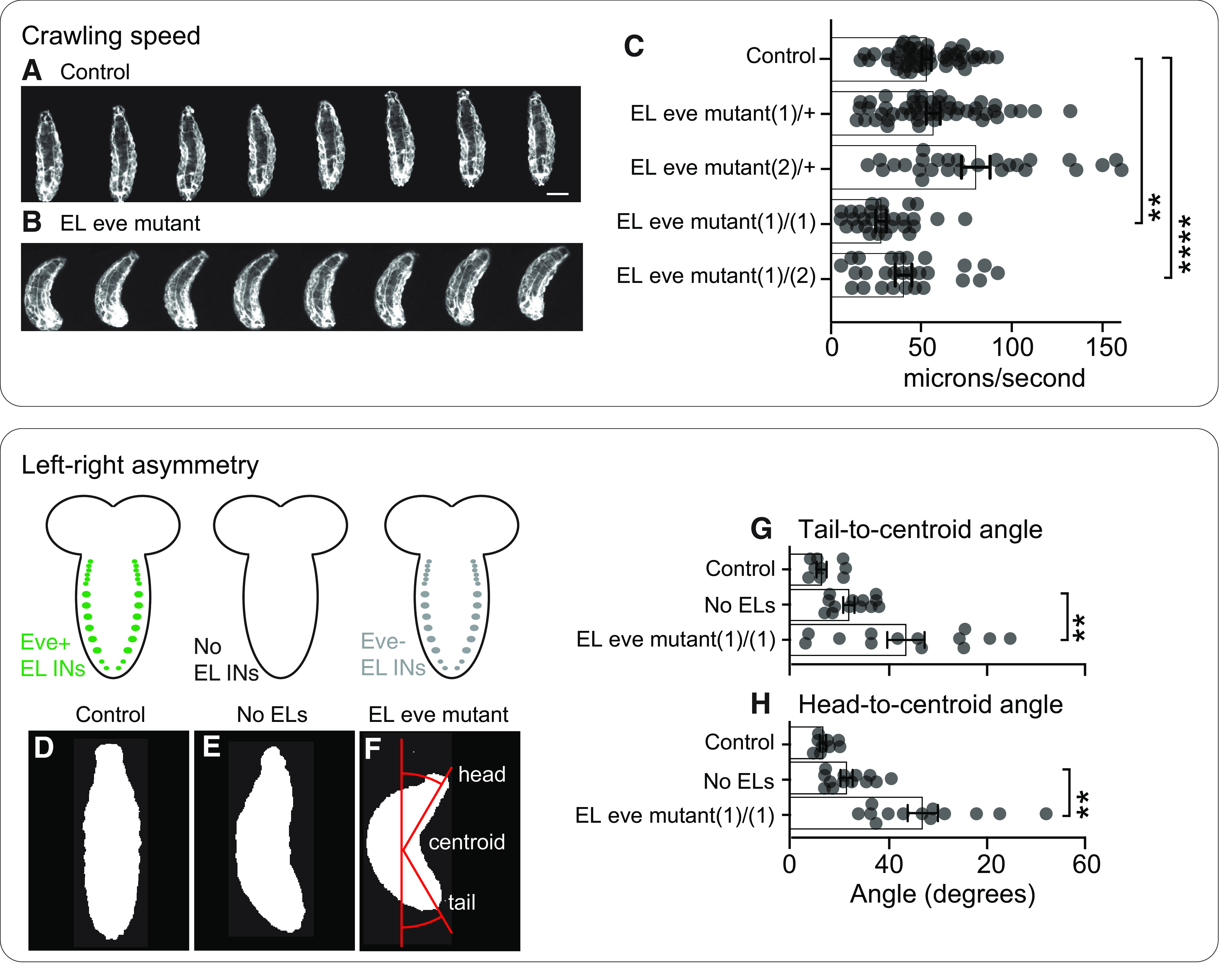
In EL eve mutants, there are defects in spontaneously-occurring crawling behavior. ***A–C***, Images and quantification of larva crawling. ***A***, ***B***, Images of control and EL eve mutants during forward crawling. Images are frames (0.66-s intervals) from representative behavioral recordings. Anterior is up and scale bar is 150 μm. C. Quantification of crawling speed is calculated as centroid movement over time. Each dot represents the average data for one larva. Bars represent average of all data points, and whiskers show SEM. One-way ANOVA with Dunnett’s multiple comparison; **p < 0.01, ****p < 0.0001. ***D–F***, Images of left-right asymmetrical body posture. ***D***, In control, larvae crawl left-right symmetrically. ***E***, When ELs are genetically ablated during embryogenesis, larvae crawl with left-right asymmetrical body posture. ***F***, In EL eve mutants, when Eve is removed from ELs, but the EL neurons remain, larvae crawl with a significantly severe left-right asymmetrical body posture. Images are single representative frames from behavioral recordings showing body shape with anterior up and scale bar of 40 μm. ***F*** is overlaid to show how angles are calculated. ***G***, ***H***, Quantification of left-right body asymmetry. Asymmetry is measured as tail-to-centroid and head-to-centroid angles during crawling. Each dot represents the average data for one larva. Data for No ELs replotted from Heckscher et al. (2015), with permission. Bars represent average of all data points, and whiskers show SEM ANOVA with Dunnett’s multiple comparison test; **p < 0.01, Genotypes: Control is wildtype (+/+); no EL is *UAS-RPR, UAS-HID/+;; EL-GAL4/+*; refer to Figure 1*D* for naming of mutant allele genotypes used in this experiment.

We used functional approaches to determine the extent to which relocation of Eve(–) EL output synapses change circuit function. We recorded spontaneously occurring crawling in control and in EL eve mutant larvae (Fig. 9). We calculated crawling speed as centroid position over time and left-right body symmetry using the angles between centroid and head position and centroid and tail position. EL eve mutants crawl significantly slower than control (Fig. 9*A–C*) with left-right asymmetrical body posture (Fig. 9*D*,*F–H*). We had previously recorded the behavior of larvae that entirely lack EL interneurons, and here we noticed that, in comparison, EL eve mutant larvae have more severe crawling defects (Heckscher et al., 2015). This is quantified as significantly greater left-right asymmetry in larvae with Eve(–) ELs than in larvae without ELs (Fig. 9*D–H**).* The discrepancy between severity of the phenotypes could be explained by the idea that mispositioned Eve(–) EL output synapses make new, functional connections, which could provide a dominant negative effect at the circuit level (see Discussion).

**Figure 10.**
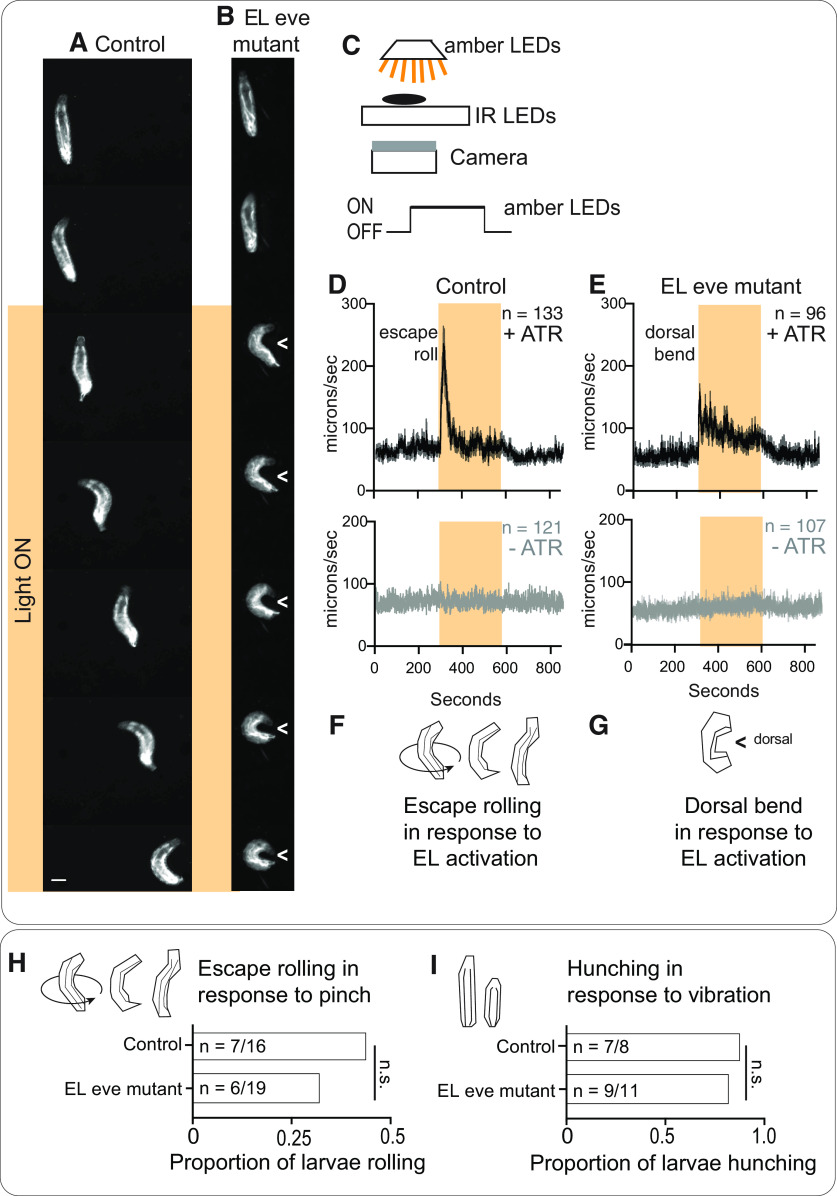
EL outputs are remapped in the absence of Eve. ***A***, ***B***, Images of behavioral responses to optogenetic stimulation of ELs. ***A***, Control larvae fed ATR and expressing CsChrimson in ELs roll in response to light. ***B***, EL eve mutant larvae fed ATR and expressing CsChrimson in ELs display a novel dorsal bend phenotype (can be either side). Images are frames from representative behavioral recordings (shown at 0.6-s intervals). Scale bar: 150 μm. In ***B***, > points to dorsal. ***C***, Illustration of the behavioral rig. The rig uses infrared light emitting diodes (IR LEDs) to illuminate larvae, which is detected by the camera, but not the larvae. Amber LEDs stimulate optogenetic effectors. ***D***, ***E***, Quantification of larval movement. Centroid speed is calculated as centroid displacement/time. Orange bar shows exposure to amber light. Gray traces (bottom) are control larvae not fed ATR, a co-factor needed for optogenetic simulation. Black traces are experimental larvae, which were fed ATR. *n* = number of larvae recorded. Average is shown as darker lines and SEM is shown as lighter lines. ***F***, ***G***, Illustrations of behavioral responses. Controls roll in response to EL activation, whereas EL eve mutants perform a dorsal bend in response to EL activation. ***H***, ***I***, Quantification of larval sensorimotor transformations. Control and EL eve mutant larvae roll in response to body wall pinch and hunch in response to vibration. Illustration of each behavior is shown in top left of each panel. For quantifications, *n* = number of larvae responding to each stimulus over the total number of larvae stimulated. Chi-squared test, n.s. = not significant. Genotype: control in ***A***, ***D*** is *UAS-FLP, act5C-FRT.stop-GAL4;* Δ*EL, Df(2R)eve/+; EL-GAL4/UAS-Cs.Chrimson.mVenus.* EL eve mutant in B and E is *UAS-FLP, act5C-FRT.stop-GAL4;* Δ*EL, Df(2R)eve/*Δ*EL, Df(2R)eve; EL-GAL4/UAS-Cs.Chrimson.mVenus*; control in ***H*** is Δ*EL, Df(2R)eve/+.* EL eve mutant in I is Δ*EL, Df(2R)eve)/*Δ*EL, Df(2R)eve*.

The crawling defects provide strong functional evidence that *eve* expression is required in EL interneurons for normal somatosensory circuit function (Fig. 9). However, we demonstrated that in Eve(–) ELs sensory encoding is disrupted (Fig. 7). Therefore, it is possible that dysfunction in sensory encoding could explain the observed behavioral deficits. And so, we assayed the behavioral response to optogenetic stimulation of Eve(–) ELs . Optogenetics uses light to activate neurons and bypasses the need for any input from upstream neurons. We reasoned that optogenetic stimulation of Eve(–) ELs might elicit a novel behavior, consistent with the idea that Eve(–) EL output synapses are remapped. In control and EL eve mutants, we expressed the optogenetic effector, CsChrimson, and fed larvae all trans retinal (ATR), a cofactor needed for CsChrimson light sensitivity. As expected, in response to light, any larva not fed ATR fails to respond (Fig. 10*D*,*E*). Also, as expected, control larvae fed ATR roll on light exposure, which is quantified as fast centroid movement over time (Fig. 10*A*,*D*,*F*). In EL eve mutants fed ATR, in response to light, larvae preform a novel behavior—a dorsal body bend. This is quantified as a small increase in centroid movement over time (Fig. 10*B*,*E*,*G*). The body bend is an extreme C shape where the head and tail nearly touch, and to our knowledge, does not occur naturally in *Drosophila melanogaster*. Dorsal bending is robust, displayed by nearly every EL eve mutant larva. This demonstrates that Eve(–) EL output synapses are functional and that their activation leads to a novel behavior.

Activation of Eve(–) EL interneurons could lead to novel behavior for two reasons. First, EL output synapses could be remapped to new, downstream targets and away from roll inducing circuits. Alternatively, circuits mediating rolling and other sensorimotor transformations could be generally dysfunctional. This possibility needed to be tested because homeodomain proteins, including Eve, can have nonautonomous effects (Lee et al., 2019). To probe the function of rolling circuits, we provided a body wall pinch, which served as a mechanical and noxious stimulus, to both control and EL eve mutants (Hwang et al., 2007). In both genotypes, larvae roll in response to pinch (Fig. 10*H*). Thus, in EL eve mutant larvae, roll inducing circuits are not disrupted. Further, we found that both EL eve mutant and control larvae respond to vibration with a hunch (Fig. 10*I*). Thus, in EL eve mutant larvae, somatosensory stimuli can be perceived and transformed into appropriate motor output, and somatosensory circuits are not generally dysfunctional. We conclude that, in Eve(–) ELs, output synapses are no longer mapped to roll inducing circuits but instead to novel targets.

## Discussion

In this study, we removed the conserved, homeodomain transcription factor, Eve, from *Drosophila* Eve-expressing ELs (Fig. 1). We found that *eve* regulates multiple aspects of EL interneuron morphogenesis (Figs. 2-4), and that *eve* is required for the proper integration of ELs into somatosensory circuits at both the input (Figs. 6, 7) and output (Figs. 8-10) levels.

### Previously undescribed roles for *eve* in neuronal morphogenesis

Here, we show that *eve* expression is required for positioning EL interneuron neurites in all three axes (i.e., medial-lateral, anterior-posterior, and dorsal-ventral; Figs. 2-4). In *Drosophila*, each axis is patterned by a separate ligand/receptor signaling system (Zlatic et al., 2009; Evans and Bashaw, 2010; Emerson et al., 2013). However, how individual interneurons read and interpret each signal is not well understood. Our data suggest *eve* is important for ELs to simultaneously read and/or interpret multiple ligand gradients simultaneously.

Generally, *eve* is considered a cell fate determinant. For example, in mouse V0v interneurons, *evx1* represses expression of *en1,* a marker of V1 interneuron identities (Moran-Rivard et al., 2001). In V0v interneurons that lack *evx1*, *en1* expression is derepressed and take on V1-like axonal projections. Similar fate changes are seen in *Drosophila* and *C. elegans* motor neurons when *eve* is disrupted (Landgraf et al., 1999; Esmaeili et al., 2002). Our data are more consistent with the idea that *eve* plays a role in the refinement of EL morphogenesis. In support for the morphogenetic refinement model is, first, in wild-type, there are no neurons with morphology that matches the morphology of Eve(–) ELs, as would be expected by a cell fate switching model. Second, there are no obvious large-scale changes in gene expression, which are typically associated with cell fate changes (Fig. 5). Third, *eve* expression in ELs is not playing a role in initial morphogenesis (see next paragraph for more).

Both Eve(–) and Eve(+) ELs cross the midline at embryonic stage 15 (Fig. 2*A–C*). Thus, *eve* expression is either dispensable for initial morphogenesis, or in EL eve mutants there is an undetectable pulse of early *eve* expression in ELs. But, we and others have not found Eve protein expression in ELs in EL eve mutants at any stage of development (Fig. 1*A*,*B*; Fujioka et al., 2003). In later stage embryos and larvae, we observe morphologic defects in Eve(–) ELs (Figs. 2*D–H*, 3, 4). This raises the possibility that, in general, *eve* genes may play a later role in morphogenesis. This is consistent with the observation that, in mouse V0v interneurons, there is early *evx1* expression and later *evx2* expression. However, the later role of *evx2* is unknown (Moran-Rivard et al., 2001).

In general, eve genes are known to regulate axon morphogenesis (Doe et al., 1988; Landgraf et al., 1999; Moran-Rivard et al., 2001; Esmaeili et al., 2002; Fujioka et al., 2003). In this study, we show that late-born Eve(–) ELs have axonal defects (Figs. 3, 4). Notably, the role of *eve* in dendrite morphogenesis is extremely poorly characterized. The distinction between dendrite and axon is important because these two compartments carry out different functions. Further, in *Drosophila*, interneuron axons and dendrites are structurally different. Dendrites are often highly branched, and lack mitochondria and postsynaptic machinery. Whereas, axon terminals (boutons) are full of mitochondria, pools of synaptic vesicles, microtubules, and vesicle release sites. Each part of the arbor (axon or dendrite) can be independently controlled by different transcription factors (Kurmangaliyev et al., 2019). For example, in *Drosophila* sensory neurons, the transcription factors Knot and Cut specifically regulate dendrite morphogenesis, but not axonal morphology (Jinushi-Nakao et al., 2007). Thus, in *Drosophila*, axon and dendrite morphology can be controlled as independent modules. Here, we show that in addition to regulating axon morphology, *eve* regulates dorsal-ventral dendrite positioning (Fig. 4). *eve* expression is also required for dendrite morphogenesis in RP motor neurons (Fujioka et al., 2003). Taken together, our data show that *eve* coordinately regulates multiple aspects of neuronal morphogenesis, and that coordinate control may be a widely-occurring role for neuronal *eve expression*.

### *eve* expression in ELs plays a role in somatosensory circuit assembly

Neuronal circuits are functional units of the nervous system. Sensorimotor circuits, specifically, transform somatosensory stimuli into motor output. Therefore, functional assays are required for the study of somatosensory circuit assembly. However, because the circuit context of individual interneurons is not well characterized, often researchers rely on anatomic assays to infer changes at the circuit level. One reason an anatomic approach can be flawed is the existence of compensatory mechanisms that allow for relatively normal circuit wiring despite changes in neuron morphology (Landgraf et al., 1999; Meng and Heckscher, 2021; Valdes-Aleman et al., 2021). In this study, we link defects in neuronal morphology to changes in circuit function, thereby explicitly demonstrating the role of *eve* expression in somatosensory circuit assembly.

#### The role of eve expression in formation of functional input synapses

We show that *eve* is required for somatosensory stimulus encoding by ELs (Fig. 7). Based on known connectivity of ELs with other neurons (Fig. 6*A*), we infer that in ELs, *eve* is required for the formation of at least four types of functional input synapses: those from vibration (chordotonal) sensory neurons to early-born ELs, from vibration-sensitive interneurons (Basins) to early-born ELs, from proprioceptive sensory neurons to late-born ELs, and from proprioceptive-sensitive interneurons (Jaams) to late-born ELs. The likely cell biological underpinning, at least for late-born ELs, is that axons from input sensory neurons (Fig. 6*G*,*H*) are not in close enough proximity to make synaptic contact with Eve(–) ELs (Fig. 4). Because of technical limitations, we could not visualize dendrite morphology of early-born ELs.

In the *Drosophila* nerve cord, there is unidirectional compensatory growth from interneurons to genetically misplaced sensory neurons (Valdes-Aleman et al., 2021; Fig. 6*C*,*D*). Thus, *Drosophila* sensory neuron-to-interneuron wiring can be robust to morphologic alterations to circuit components. The observation (Fig. 6*G*,*H*) that sensory neurons do not grow to reach mispositioned Eve(–) EL dendrites raises two possibilities: (1) in this system, compensatory growth is unidirectional (i.e., interneurons grow to misplaced sensory neurons, but not vice versa); and (2) alternatively, compensatory growth is bidirectional, however, *eve* expression is required for this process. Future experiments will be needed to distinguish between these models.

#### The role of *eve* in positioning output synapses

Our data show Eve(–) EL output synapses are functional, but remapped. Spontaneously-occurring crawling behavior is disrupted in EL eve mutants (Fig. 7), and that this disruption is significantly worse than in larvae which lack EL neurons altogether. This could be explained by requirement for ELs during early circuit development (e.g., acting as a scaffold for normal axonal pathfinding for other neurons). Alternatively, mature Eve(–) ELs could exert a dominant negative effect at the level of circuit function. We favor the latter idea because it is consistent with the optogenetic experiments presented in Figure 9, and the anatomic data presented in Figure 7. Figure 7 shows that in controls, EL output synapses are excluded from many zones of the neuropile including the dorsal lateral zone, which houses the dendrites of dorsally-projecting motor neurons. However, Eve(–) ELs are likely to form output synapses in this region. This specific re-distribution of output synapses is notable because it raises the possibility that Eve(–) ELs output synapses (ELs are excitatory) could be directly re-mapped to dorsal motor neurons. Such a re-mapping could explain the novel behavioral phenotype, dorsal body bending phenotype seen on optogenetic activation of Eve(–) ELs in Figure 9. Regardless of the exact anatomic changes, the data in Figure 9 show that output synapses of Eve(–) ELs are functional, but are functionally re-mapped to new output circuits.

In conclusion, we have provided an updated understanding of the role of *eve* expression in neurons. Our data provide understanding of the role of neuronal *eve* at the levels of circuit physiology and animal behavior. Further they provide insight into the genetic logic of somatosensory circuit assembly, demonstrating that multiple terminal neuronal features can be coordinately regulated by the activity of a single postmitotic transcription factor. Finally, our data raises new questions about the role of *eve* expression in other neuron types and enable future experimental inquiry into somatosensory circuit assembly in *Drosophila*.

## Synthesis

Reviewing Editor: Miriam Goodman, Stanford University Decisions are customarily a result of the Reviewing Editor and the peer reviewers coming together and discussing their recommendations until a consensus is reached. When revisions are invited, a fact-based synthesis statement explaining their decision and outlining what is needed to prepare a revision will be listed below. The following reviewer(s) agreed to reveal their identity: NONE. Note: If this manuscript was transferred from JNeurosci and a decision was made to accept the manuscript without peer review, a brief statement to this effect will instead be what is listed below.

Thank you for transferring your manuscript to eNeuro, based on the reviews you have already received and the revisions provided in response to these critiques, we will be able to take a decision without additional review. Following a detailed reading of the revised manuscript, please consider taking the following actions in a second revision:

1. Re-integrate the negative results of screening other candidate transcription factors. Doing so will enable readers to gain a fuller view of the role of eve and other transcription factors in the morphology and function of EL neurons. This action is not necessary for publication, but it is my editorial opinion that it would enhance the impact of the study.

2. The manuscript has several run-on sentences as well as sentence fragments that impair readability. It will benefit from additional editing and revision. To assist you, I am attaching a marked up copy of your submitted manuscript identifying specific areas that will benefit from revision. You need not accept all of these
